# Comparison of Two Entry Methods and Their Cosmetic Outcomes in Creating Pneumoperitoneum: A Prospective Observational Study

**DOI:** 10.1055/s-0042-1756182

**Published:** 2022-09-02

**Authors:** Srikantaiah Chandra Sekhariah Hiremath, Zameer Ahmed

**Affiliations:** 1 Department of General Surgery, M. S. Ramaiah Medical College, Bengaluru, Karnataka, India

**Keywords:** prospective study, pneumoperitoneum, laparoscopic surgery, Veress technique, Hasson technique, cosmetic outcomes

## Abstract

**Background** The main challenge in laparoscopic surgery is creating pneumoperitoneum using various surgical techniques. Every procedure has its own advocates. The aim of this study was to determine the cosmetic outcomes of the two of the major surgical techniques (open—Hasson technique versus closed—Veress technique) used in laparoscopic surgery.

**Methods** This was a prospective, observational, comparative study conducted from October 2017 to September 2018 in 132 patients, who presented to our center and fulfilled our selection criteria. For all the patients, pneumoperitoneum was performed using either open (Hasson) or closed technique (Veress). A database was created for all the patients and the technique dependent cosmetic outcomes were assessed and reported.

**Results** There were a total of 66 patients in each group (open and closed). The mean age of the open group was 51.56±11.42 years and closed group was 54.36±14.78 years, respectively. The major comorbidities found in both the groups were diabetes mellitus (6/66, group A; 7/66, group B) and hypertension (3/66, group A; 4/66, group B). In open group, umbilical (58/66, *p* =0.001) and in closed group infraumbilical (35/66, *p* =0.001) were the most commonly used incisions.

**Conclusion** As benefits outweigh the risks, the better cosmetic outcomes were observed in patients underwent closed technique over open technique ( *p* <0.05).

Laparoscopy (from Greek lapara, “flank or loin” and skopein, “to see, view or examine) is the art of distending and examining the abdominal cavity by air through a procedure called “pneumoperitoneum.” Such procedure is also called as “keyhole surgery” or “minimal invasive surgery” in modern surgical terminology. In 1901, for the first time this technique was introduced and used by Georg Kelling on dogs. 1 Followed by in 1910, Hans Christian performed the first laparoscopic surgery in humans. 1 2 Since then, it evolved constantly and emerged as a preferred surgical option for a multitude of operative, therapeutic, and diagnostic purposes. 3 Compared with traditional laparotomy (open surgery), laparoscopic technique (closed surgery) has several advantages such as larger field of surgery, minimal traumatic insult, quick postoperative recovery, reduced overall risk and hospitalization time, reduced postsurgical pain and associated stress, cost-effectiveness, and improved cosmetic outcomes. 3 4 5 6 7

Despite technical advancement in laparoscopic procedures, entry and establishment of pneumoperitoneum are still a complicated process causing bowel perforations (0.1–0.2%), vascular injuries (0–0.2%), with a mortality rate of 3.3 per 100.000 in rare cases. 3 5 8 9 The major problem involved with these procedures is their postoperative injuries. In many instances, patients will present with signs and symptoms of intra-abdominal abscesses and peritonitis on follow-up. In rare cases, patients present with minor complications such as carbon dioxide embolism (0.001%), hepatic injury, and urologic injury. 10 11 12 13 14

As of date in the literature, many techniques, methods, and instruments have been described and studied to know the best possible method to minimize the surgically associated complications and none were proven to be universally effective. Such surgical techniques include Hasson technique (HT, open), Veress needle technique (VNT, closed), radially expanding trocars, disposable shielded trocars, direct trocar insertion, and visual entry systems. However, in the end, the choice of surgical technique to be opted is entirely dependent on the patient's condition, surgeon's preference, surgical skills, good knowledge of the instrumentation, technology, and other local/or regional factors. Today, some 30 years on, the debate still continues and no consensus was reached regarding the best method of gaining access to the peritoneal cavity without much postoperative complication and cosmetic outcomes. To assess such cosmetic outcomes, five different scar scales were popularly used in the literature: Stony Brook Scar Evaluation Scale, visual analog scale, Patient and Observer Scar Assessment Scale, Manchester Scar Scale, and the Vancouver Scar Scale (VSS). 15 Among all the scales, we have considered VSS for subjective scar assessment for this study. 15 16 17

Most of the present available literature is into creation of the pneumoperitoneum and studying its related complications. However, studies/literature related to cosmetic outcomes of the surgical techniques especially the most widely used open and closed procedures are observed to be very limited. In this study, to know such procedural cosmetic outcomes of two such main surgical techniques that are widely used in achieving better pneumoperitoneum, HT and VNT were studied and discussed in detail. 1 3 4 5 6 7 9 11 14 18 19 20 21 We have also compared and evaluated the cosmetic outcome of primary port insertion at the umbilicus in both the techniques.

## Materials and Methods

This prospective, randomized, observational study was conducted in 132 patients who underwent laparoscopic surgery by either HT or VNT. The study was conducted at M. S. Ramaiah Medical College and Hospitals, India, between October 2016 and September 2018 after procuring all the approvals from Institutional Ethical Committee (SS-1/EC/26/2016).

### Inclusion Criteria

All the patients presented to our center with complaints of acute or chronic abdominal pain requiring surgical intervention with particular emphasis on diagnostic/therapeutic laparoscopy for hernia, appendix, and gallbladder were included.

### Exclusion Criteria

Patients outside the purview of general surgery were excluded, which include cases of obstetrics-gynecology, large paraumbilical hernia, incisional hernia, acute infective surgical condition, dermatological condition, midline vertical laparotomy scar, and history of abdominal surgeries. Other medical conditions include liver cirrhosis, coagulopathy, and international normalized ratio (INR)—INR more than 1.4.

### The Vancouver Scar Scale

The VSS was first introduced in 1990 and it has been used extensively in literature since then to determine the various factors such as scar height or thickness (0—normal: flat, 1—<2mm, 2—<5mm, 3—>5mm), vascularity (0 normal color that closely resembles the color over the rest of one's body, 1—pink, 2—red, 3—purple), pigmentation (0—normal color that closely resembles the color over the rest of one's body, 1—hypopigmentation, 2—hyperpigmentation), and pliability (0—normal; 1—supple: flexible with minimal resistance; 2—yielding: giving way to pressure; 3—firm: inflexible, not easily moved, resistant to manual pressure; 4—banding: rope-like tissue that blanches with extension of the scar; 5—contracture: permanent shortening of scar producing deformity or distortion). 15 16 17 22 The VSS scoring from these factors was ranged between 0 and 13 points and based on the final score, the subjective scar assessment was done. 15 23

### Statistical Analysis

The VSS was used to analyze the following parameters—scar vascularity/pigmentation/pliability/height. Continuous variables were presented as mean for parametric data and as median, if the data was non parametric or skewed. Student's *t* -test was applied for data following normative distribution and Mann–Whitney U test for non-normative distribution. Categorical variables were expressed as frequencies and percentages. Nominal categorical data between the groups was compared using chi-squared test or Fisher's exact test, as appropriate. *p* -Value less than 0.05 was taken to indicate a statistically significant difference. Minitab version 17 (Minitab, LLC., State College, PA) was used for computation of statistics.

## Results

**Fig. 1:**
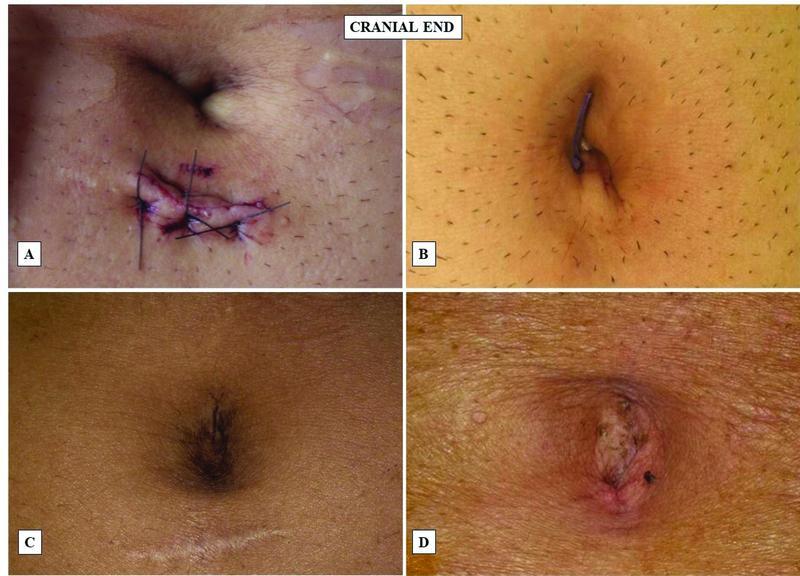
The cosmetic outcome of the primary port insertion at the umbilicus in both open technique (Hasson, A and C) and closed technique (Veress, B and D) in laparoscopic surgery.

Of the enrolled 132 patients, both the groups (VNT, group A and HT, group B) were randomized with 66 patients each. As shown in Table 1 , majority of the patients were ranged between the age of 20 and 40 years ( *p* =0.43) with a preponderance toward male (36/66, group A; 34/66, group B) over females in both the groups. Majority of the patients have a history of malignancy (6/66, group A; 5/66, group B), coagulopathy (4/66, group A; 3/66, group B) with comorbidities such as diabetes mellitus (6/66, group A; 7/66, group B) and hypertension (3/66, group A; 4/66, group B). The presenting symptoms in most of the patients were abdominal pain (42/66, group A; 37/66, group B), vomiting (26/66, group A; 22/66, group B), and swelling (12/66, group A; 15/66, group B). However, with the surgical incisions opted, umbilical in group A (58/66, *p* =0.001) and supraumbilical (31/66, *p* =0.001) in group B are the most widely opted incisions in both the groups. The most used suture material during the surgical procedures in both the groups was nylon and staples was used in only one patient of the group B. The postoperative assessment of scars and cosmetic outcomes were assessed by using VSS scale and its data was presented in Table 2 , Fig. 1A–D .

**Table 1:** Baseline demographics and other variables of the patients underwent surgery using Veress technique and Hasson technique

Variables	Veress technique (Group A)		Hasson technique (Group B)		*p* -Value (chi-squared test)
	No. of patients ( *n* =66)	Percentage (%)	No. of patients ( *n* =66)	Percentage (%)	
**Age group (years)**					
< 20	7	10.61	8	12.12	0.43
20–30	16	24.24	19	28.79	
30–40	12	18.18	9	13.64	
40–50	17	25.76	16	24.24	
50–60	14	21.21	14	21.21	
**Gender**					
Male	36	54.55	34	51.52	–
Female	30	45.45	32	48.48	–
**Patient history**					
Previous Sx	2	3.03	3	4.55	0.77
Trauma	3	4.55	2	3.03	0.64
Malignancy	6	9.09	5	7.58	0.81
Coagulopathy	4	6.06	3	4.55	0.92
**Comorbidities/treatment**					
Diabetes mellitus	6	9.09	7	10.61	0.74
Hypertension	3	4.55	4	6.06	0.82
Liver disease	2	3.03	3	4.55	0.72
On steroids	1	1.52	1	1.52	0.84
**Symptomology**					
Abdominal pain	42	63.64	37	56.06	0.41
Vomiting	26	39.39	22	33.33	0.32
Swelling	12	18.18	15	22.73	0.46
**Comparison of incision**					
Umbilical	58	87.88	0	0	0.001
Supraumbilical	2	3.03	31	46.97	0.001
Infraumbilical	6	9.09	35	53.03	0.001
**Suture material**					
Nylon suture	66	100	65	98.48	0.87
Staples	0	0	1	1.52	
**Diagnosis**					
Appendicectomy	28	42.42	23	34.85	0.64
Inguinal hernia	26	39.39	25	37.88	
Cholelithiasis	12	18.18	18	27.27	

**Table 2:** Assessing and rating the postsurgical scars of patients from both groups using Vancouver Scar Scale

Variables	Veress technique	Hasson technique	*p* -Value (Student's *t* -test)
Pigmentation	0.54±0.65	0.62±0.57	0.041
Vascularity	0.58±0.67	0.68±0.71	0.023
Pliability	0.64±0.75	0.76±0.62	0.034
Height	0.52±0.58	0.56±0.64	0.021
Total score	2.28±0.64	2.62±0.63	0.026

## Discussion

Laparoscopic surgery is evolving into a well-established procedure over the years by improving itself in terms of instrumentation, technicality, and guidelines. But still it has been observed as a controversial procedure in terms of creating a pneumoperitoneum. To develop a safest pneumoperitoneum entry/or establishing, a safest technique among available multiple entry techniques is still a debate. The selection of the best suitable entry technique for any patient is completely dependent on the patient's condition and their interest on cosmetic outcomes. In this study, we have explored the most commonly used entry techniques (open, HT and closed, VNT) with special focus on cosmetic outcomes. In both the techniques, no notable complications related to vascular or bowel injury were reported during the creation of pneumoperitoneum.

However, in open technique (HT), minor concerns such as bowel injury, gas leaking, improper cosmetic outcome, and long surgical were was reported. However, in the closed technique (VNT), postoperative complications were a concern but the benefits associated with it have outweighed the risks in terms of cosmetic outcomes and reduced postoperative trauma. In the end, postoperative subjective scar assessment and cosmetic outcomes were assessed in both the groups using VSS scale and results have suggested VNT as a better technique over HT ( Fig. 1A–D ). 15 23

Such difference in extent of scar and the cosmetic outcomes between both the procedures was also observed to be highly dependent on multiple other factors such as (a) type of incision and (b) the preferred entry route (infraumbilical or supraumbilical or transumbilical). Where in our study group, almost all the HT patients underwent transumbilical incision and VNT patients—supraumbilical or infraumbilical incision. As of date, very few studies are available in the literature in terms of the cosmetic outcomes based on the incision type and the entry route taken. Unfortunately, no clear consensus was drawn from these studies in terms of best incision technique to be considered for better cosmetic outcomes. However, reports from our study have shed some light in the present gray area by reporting some positive outcomes in patients who underwent VNT technique over HT.

Findings from our study were in line with multiple previous studies from the literature supporting both supraumbilical and transumbilical as a choice of incision 24 25 26 27 for better cosmetic outcomes with nearly normal looking umbilicus postoperatively. On the contrary, Sasmal et al 28 have reported vertical incision as a better alternative over transverse incision for better postoperative cosmetic outcomes. In a large prospective study conducted by Şentürk et al has reported some other contradicting results by showing no difference between the supra-trans-infraumbilical incisions and their related cosmetic outcomes. 24

Overall in a nutshell, from our experience and from the literature, it was observed that cosmetic outcomes are not only entirely dependent on the type of incision taken but they are also highly dependent on multiple patient-related variables such as age, obesity, and comorbidities, where age is inversely proportional to the wound healing process due to the reduced skin elasticity. However, comorbidities such as diabetes and obesity can cause negative scar healing with high risk of infection due to relatively insufficient nutritive blood supply to adipose tissue. In patients with liver diseases, quality of elastogenesis is reported to be poor, leading to poor quality of scar. In coagulopathy patients, hematomas and its associated infections lead to poor healing process. 29 Another major concern is postsurgical pigmentation in these patients due to the deep surgical injuries, where all the adnexal elements were removed or destroyed causing hypopigmented centers in contrast to the surrounding unwounded skin. 30 31 32 33 Whereas with the pliability HT is less supple than normal skin due to thick scar and inferior quality of collagen architecture, leading to skin deformation and decreased skin elasticity, stiffness, and laxity. Overall, multiple mechanisms, patient-related variables, and various other dependent, and independent factors were involved in terms of scaring, healing, and final cosmetic outcomes of the patient.

However, the single-center nature and low number of patients can be a major limitation of this study, where its results cannot be widely generalized.

## Conclusion

The findings of our study suggest that for intraperitoneal access in laparoscopy, both the HT and VNT were observed to be safe. As of date, no study has clearly demonstrated the superiority of one entry technique over other. Overall from our study we observed that VNT is a good alternative for pneumoperitoneum creation in laparoscopic surgeries over HT due to its relatively low entry-related injuries and better cosmetic outcomes. However, clinicians should understand that no single technique is considered suitable for all the cases and in the end it is largely dependent on patient's demographics, and intrinsic characteristics (medical history, comorbidities, etc.). To conclude, the choice of technique for peritoneal access for better cosmetic outcomes can be VNT over HT. Further large-scale prospective studies are needed at multiple centers and on larger samples for conclusive evidence.
